# Supplementary figures and images for: Inka2, a novel Pak4 inhibitor, regulates actin dynamics in neuronal development
Source: PLoS Genet. 2022 Oct 27;18(10):e1010438. doi: 10.1371/journal.pgen.1010438 (PMC9612522; doi:10.1371/journal.pgen.1010438)

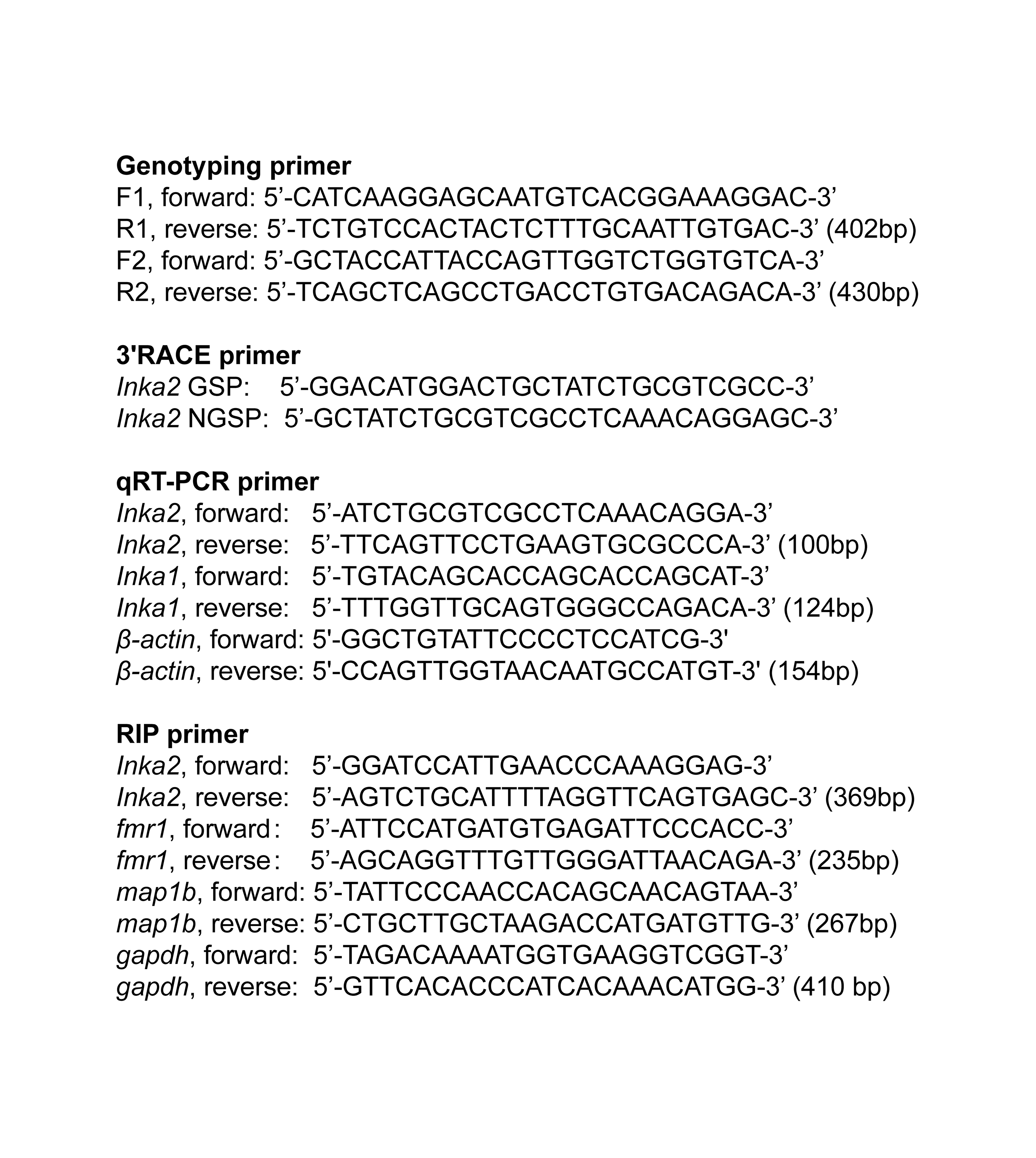

Supplement: S1 Table — (TIF) [file pgen.1010438.s001.tif]

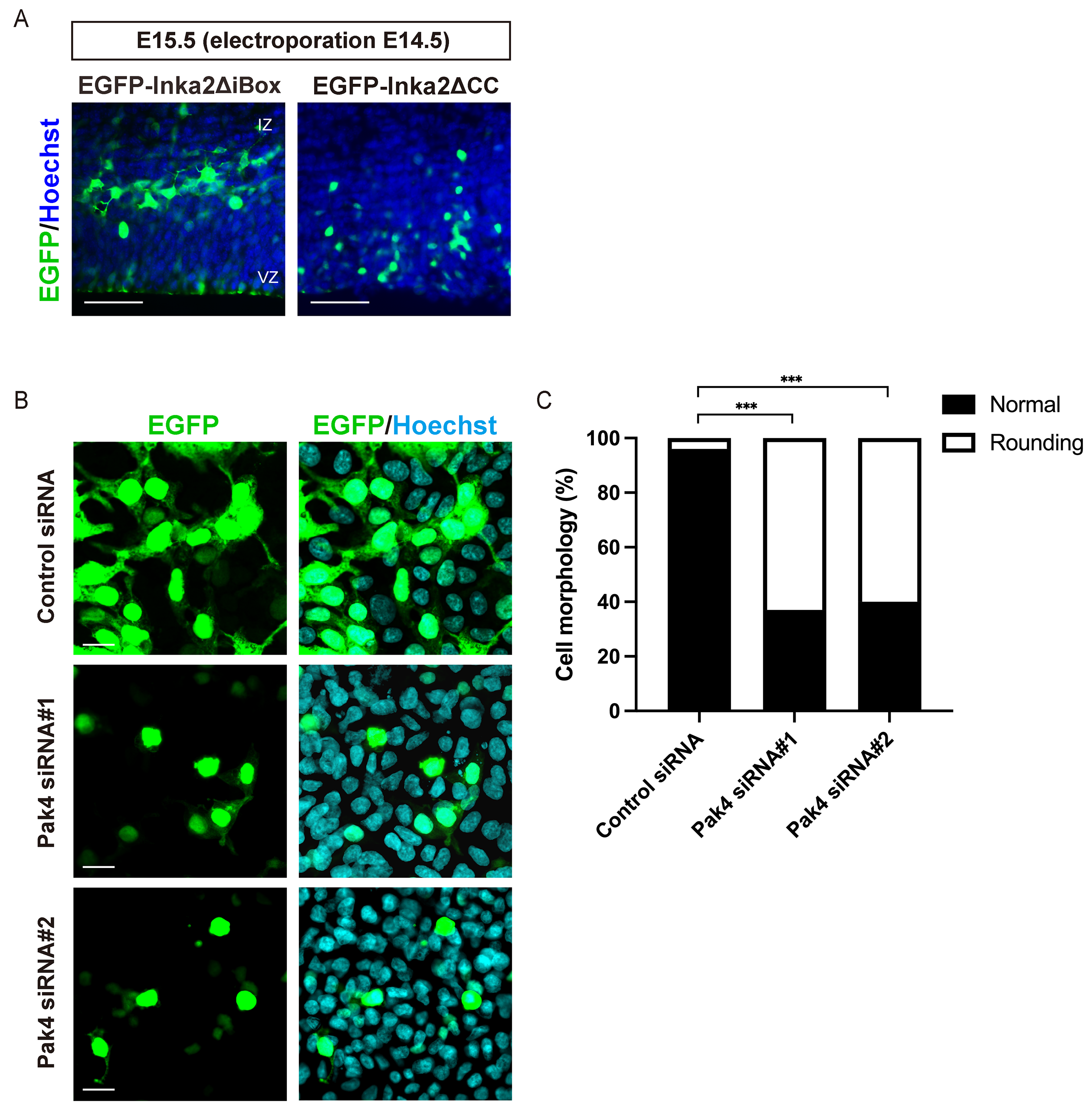

Supplement: S1 Fig — (A) In utero electroporation of CAG-EGFP-Inka2ΔiBox or CAG-EGFP-Inka2ΔCC was performed on E14.5, and the neocortex was analyzed at E15.5. Nuclei were counterstained with Hoechst dye (blue). VZ, ventricular zone; IZ, intermediate zone. (B, C) HEK293T cells were transfected with non-targeting control siRNA or two different Pak4 siRNAs (#1 and #2) along with CAG-EGFP. (C) Quantified comparison of the effect of Pak4 knockdown on cell morphology. The stacked bar chart shows the proportion of cells that exhibited a normal or rounded morphology. ***, P < 0.001; chi-square test with Holm–Bonferroni correction. Control, n = 367 cells; Pak4 siRNA#1, n = 198 cells; Pak4 siRNA#2, n = 211 cells. Scale bars, 50 μm in (A) and 20 μm in (B). (TIF) [file pgen.1010438.s002.tif]

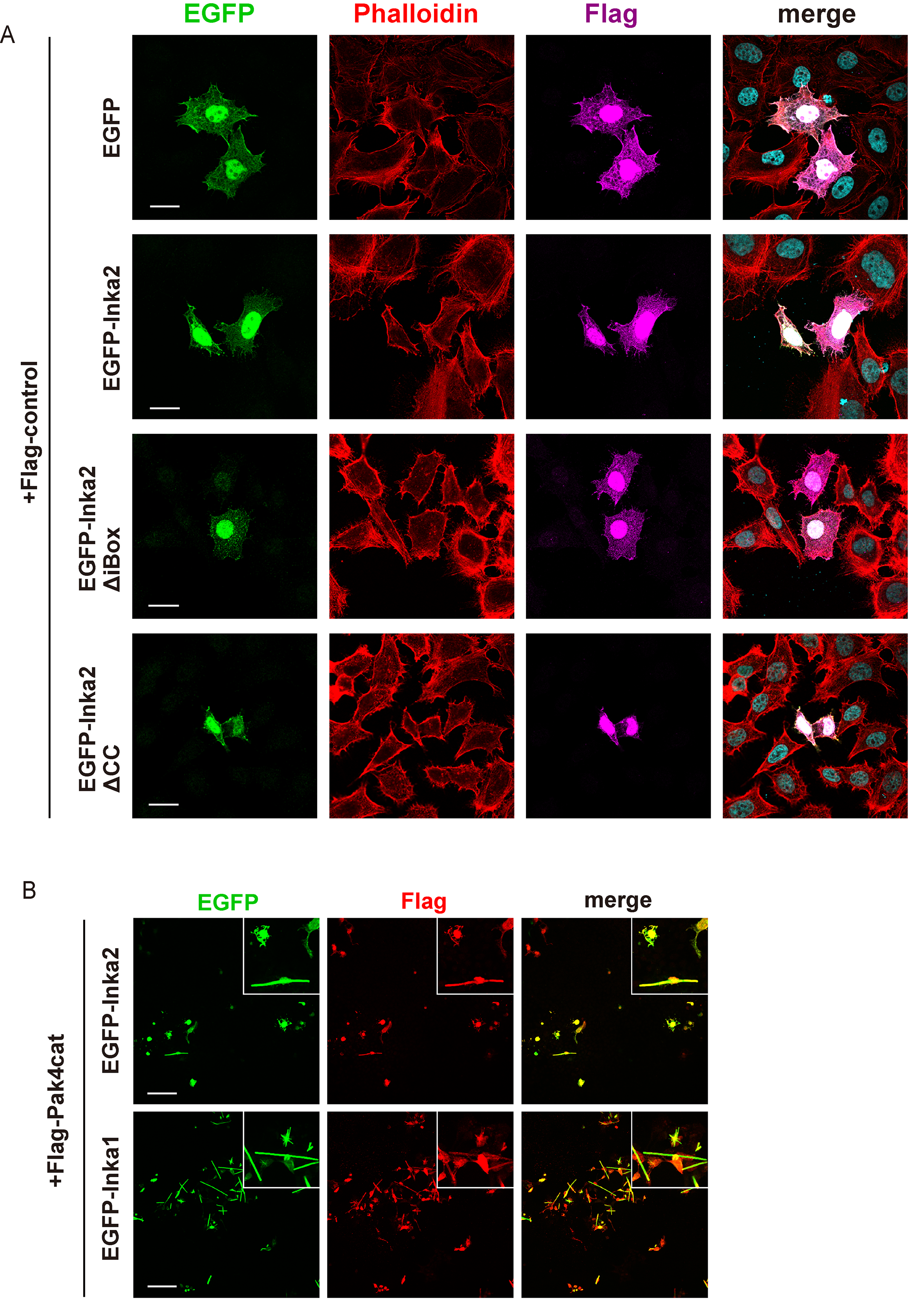

Supplement: S2 Fig — (A) HeLa cells were transfected with control EGFP, EGFP-Inka2, EGFP-Inka2ΔiBox, or EGFP-Inka2ΔCC along with Flag-control. Cells were immunostained with anti-Flag antibody (magenta), and F-actin was stained with phalloidin (red). (B) HEK293T cells were transfected with control EGFP-Inka2 or EGFP-Inka1 along with Flag-Pak4cat. Cells were immunostained with an anti-Flag antibody (red). Insets show a magnified view of the needle-shaped protein crystals growing within the individual cell. Simultaneous expression of EGFP-Inka1 and Pak4cat often caused in cellulo crystallization of the protein complex containing Inka1. Scale bars, 20 μm in (A) and 100 μm in (B) (TIF) [file pgen.1010438.s003.tif]

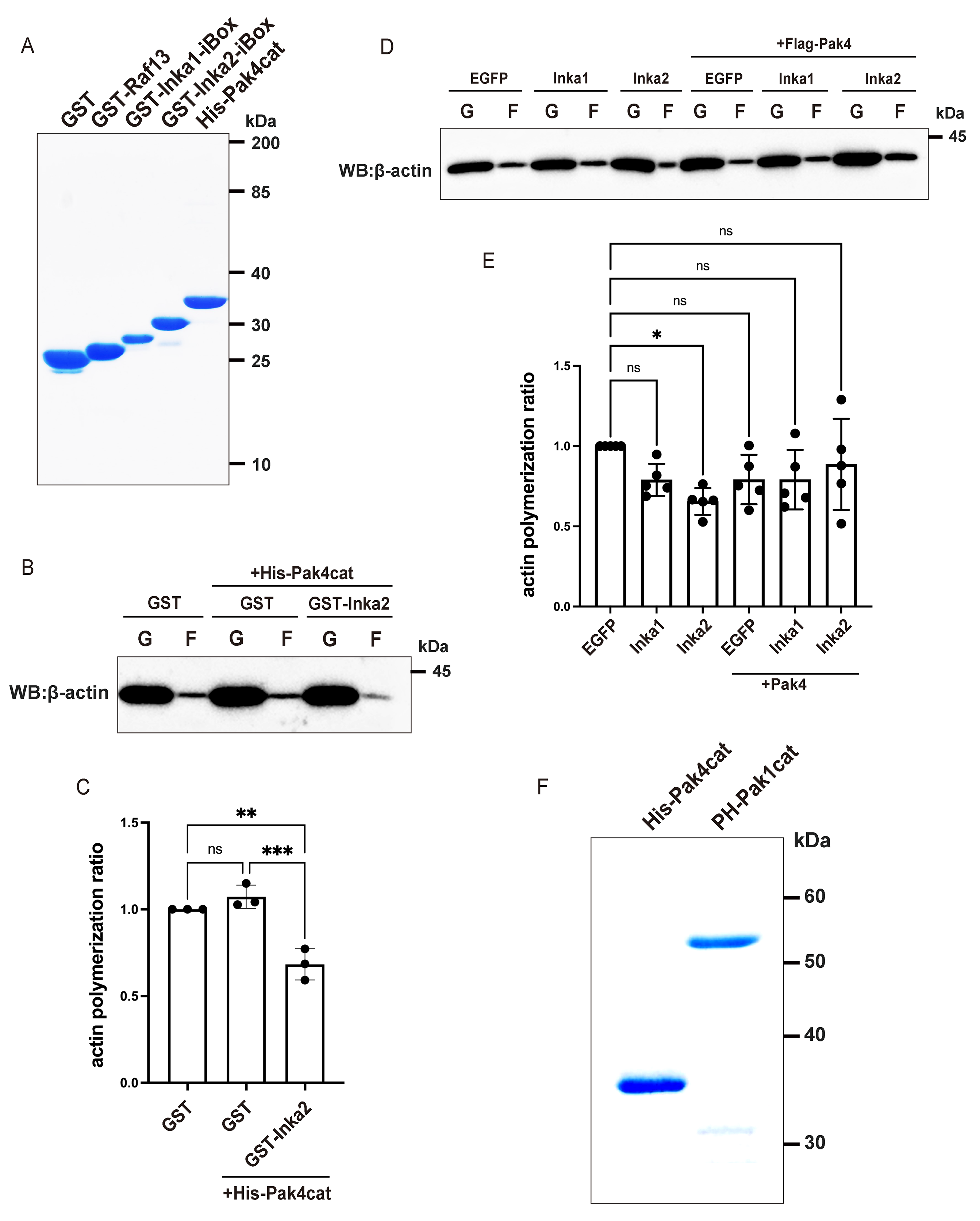

Supplement: S3 Fig — (A) Purified recombinant proteins of GST, GST-Raf13, GST-Inka1-iBox, GST-Inka2-iBox, and His-Pak4cat. SDS-PAGE and Coomassie brilliant blue (CBB) staining confirmed the quality of each protein. (B, C) Cell lysate prepared from HEK293T cells was incubated with the purified proteins of GST or GST-Inka2-iBox together with His-Pak4cat. After incubation, F-actin and G-actin fractions were separated and quantified by immunoblotting with an anti-β-actin antibody. (C) Quantified analysis of actin polymerization (F-actin/G-actin) ratio of three independent experiments. ns, not significant, *, P < 0.05; One-way ANOVA. Holm–Sidak’s multiple comparisons test. (D, E) HEK293T cells were transfected with EGFP, EGFP-Inka1, and EGFP-Inka2, together with Flag-Pak4. Fractions of F-actin and G-actin were separated by ultracentrifugation from the cell lysates and quantified using immunoblotting with an anti-β-actin antibody. (E) Quantified analysis of the actin polymerization (F-actin/G-actin) ratio of five independent experiments. ns, not significant, *, P < 0.05; One-way ANOVA. Holm–Sidak’s multiple comparisons test. (F) Purification of Pak4cat and Pak1cat. Bacterially expressed and affinity-purified recombinant proteins of His-Pak4cat and Pros2-His-Pak1cat (PH-Pak1cat). The quality of the purified proteins is confirmed by SDS-PAGE and CBB staining. (TIF) [file pgen.1010438.s004.tif]

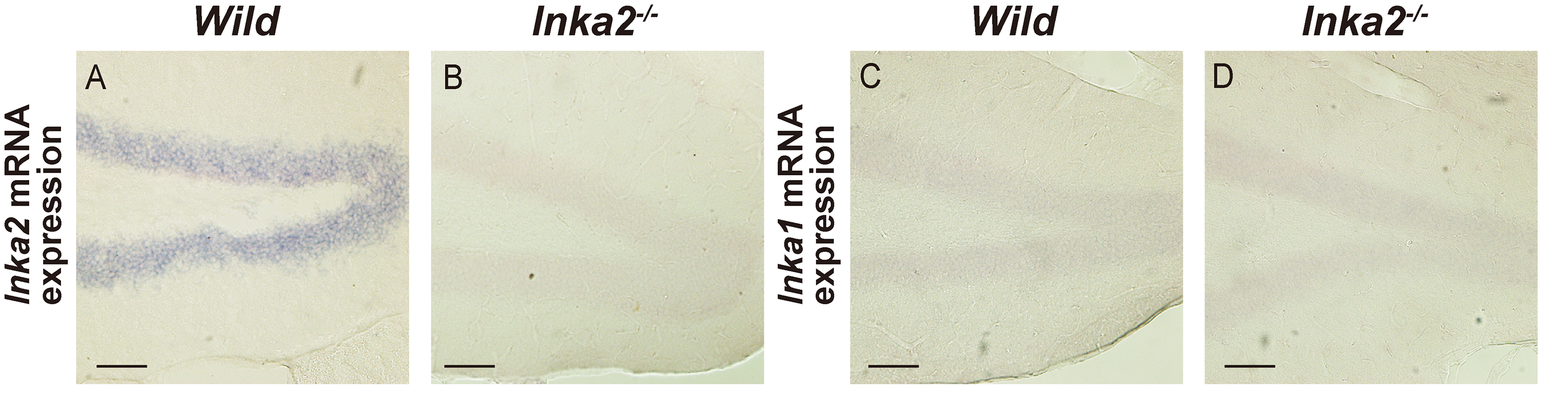

Supplement: S4 Fig — (A–D) In situ hybridization of Inka2 (A, B) and Inka1 (C, D) mRNAs in the dentate gyrus of wild-type (A, C) or Inka2-/- adult mice (B, D). Scale bars, 50 μm in (A–D). (TIF) [file pgen.1010438.s005.tif]

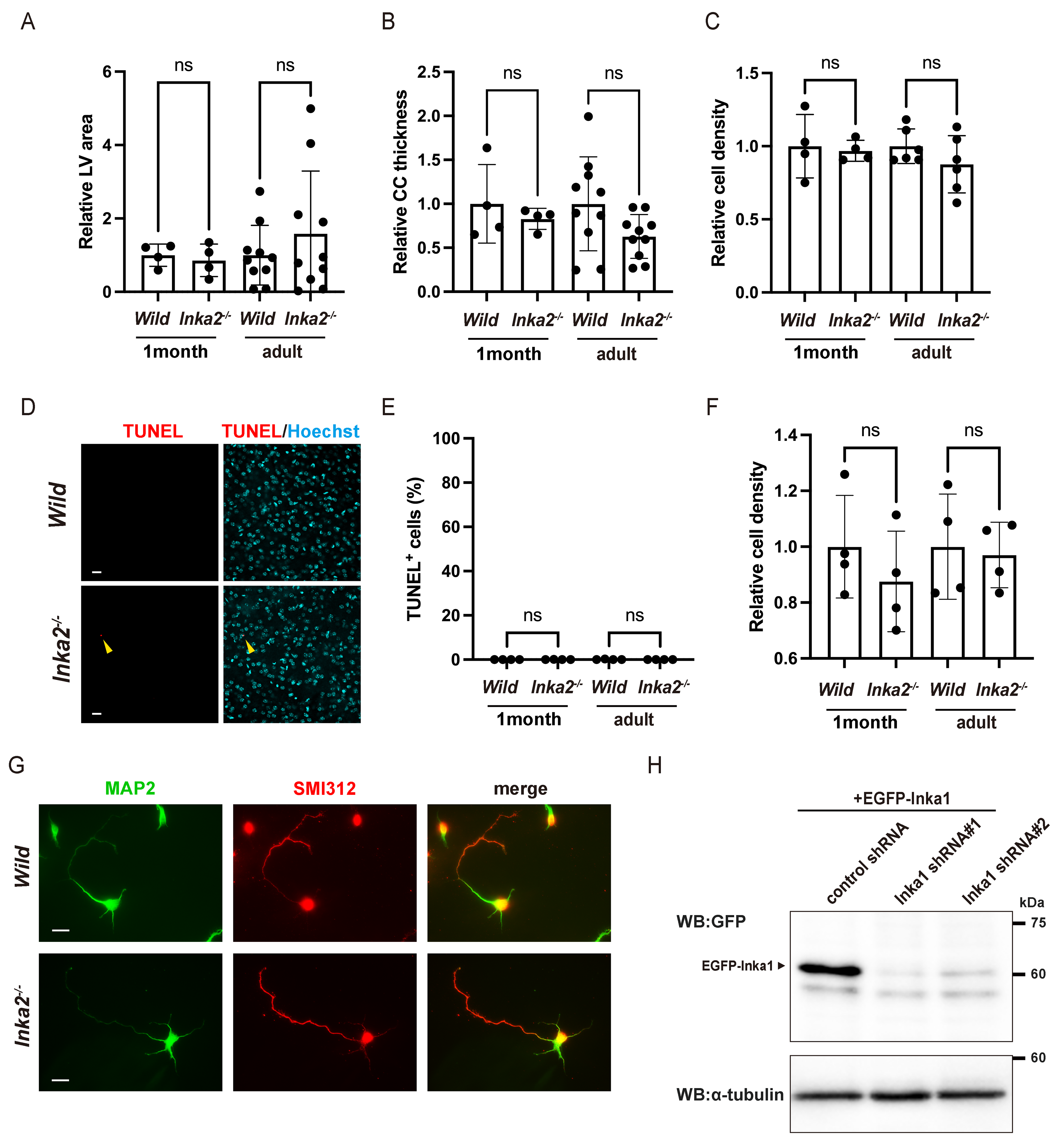

Supplement: S5 Fig — (A, B) Quantification of LV area (A) and CC thickness (B) of one-month-old and adult mice brains. ns, not significant; Welch’s t-tests with Holm–Bonferroni correction. (C) The number of unaltered neurons in Inka2-/- cerebral cortices. Cell density was determined using Nissl staining in cortical layer V of wild-type and Inka2-/- mice, and was measured at one and 12 months of age. ns, not significant; Welch’s t-tests with Holm–Bonferroni correction. (D) Apoptotic cells in the adult mice cerebral cortex were detected by TUNEL staining (red). Nuclei were counterstained with Hoechst dye. Arrowhead indicates TUNEL+ cell. (E) Quantified comparison of TUNEL+ apoptotic cells in one-month-old and adult mice cortices. ns, not significant; Welch’s t-tests with Holm–Bonferroni correction. (F) The cell density of one-month-old or adult cerebral cortices. The brain sections of wild-type or Inka2-/- mice were stained with Hoechst dye, and the cell number within cortical layer V was counted. ns, not significant; Welch’s t-tests with Holm–Bonferroni correction. (G) PCNs dissociated from wild-type or Inka2-/- embryonic cortices were cultured and then double-immunostained with anti-MAP2 and anti-SMI312 antibodies at 3 div. (H) Validation of Inka1 shRNAs. The non-targeting control or Inka1 shRNA (shRNA #1 or shRNA #2) was transfected into HEK293T cells expressing EGFP-Inka1. Subsequently, each cell lysate was subjected to immunoblotting with anti-GFP and anti- α-tubulin antibodies. Scale bars, 20 μm in (D) and (G). (TIF) [file pgen.1010438.s006.tif]

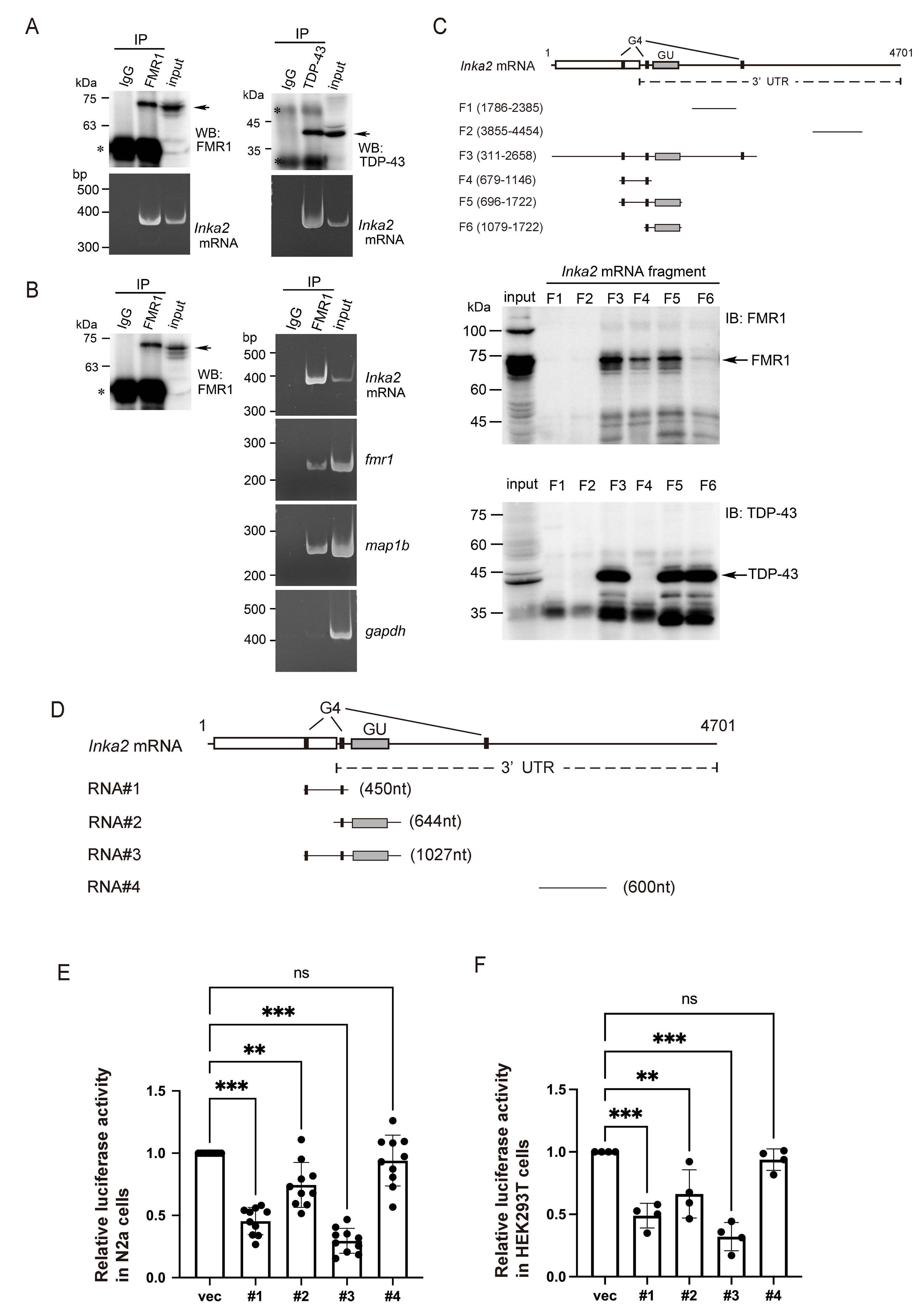

Supplement: S6 Fig — (A, B) RNA-immunoprecipitation (RIP) analysis showing the interaction between Inka2 mRNA and the RNA-binding proteins FMR1 and TDP-43. Cell lysate from N2a cells (A) and adult mouse cerebral cortex (B) were subjected to immunoprecipitation (IP) with an anti-FMR1 or anti-TDP-43 antibodies to purify the ribonucleoprotein complex containing FMR1 and TDP-43. Inka2 mRNA was detected by RT-PCR. The lower panels in A and the right panels in B represent the RT-PCR products separated by agarose gel electrophoresis. As FMR1 binds map1b mRNA, the map1b and gapdh mRNAs were used as a positive and negative control, respectively. The upper panels in A and the left panels in B show the immunoblot of IP and input lysate. (C) RNA pull-down assay using the Inka2 mRNA fragments containing G4 or GU-repeat element. The schematic diagram represents the three G4s (filled square) and GU-repeat element (gray rectangle) in Inka2 mRNA. An open square indicates the Inka2 ORF. The indicated Inka2 cDNA fragments (F1–F6) were in vitro transcribed for RNA pull-down assay. Each BrU-labeled Inka2 RNA was incubated with N2a cell lysate, followed by IP with anti-BrdU antibody to precipitate the RNA fragment–protein complexes. Immunoprecipitated proteins were analyzed by immunoblotting with anti-FMR1 or anti-TDP-43 antibodies (lower panels). (D–F) Luciferase reporter assay, showing the translational repression by the Inka2 mRNA elements. (D) Depicted regions of Inka2 mRNA (#1–#4) were inserted downstream of the firefly luciferase gene. (E, F) Each dual-luciferase construct pmirGLO-Inka2 mRNAs (#1–#4) was transfected to N2a (E) or HEK293T (F) cells, and the luciferase activity was measured. Firefly luciferase activity was normalized by the hRenilla luciferase activity and represented as the relative luminescent units (RLU). Five (for HEK293T) and 10 (for N2a) independent experiments were performed. ns, not significant; **, P < 0.01; ***, P < 0.001; One-way ANOVA. Weltch’s t-test with Holm–Bonferroni cor [file pgen.1010438.s007.tif]
